# Genome sequence of *Pyrococcus woesei* DSM 3773^T^—an anaerobic, hyperthermophilic archaeum from heated marine solfataras

**DOI:** 10.1128/mra.00528-25

**Published:** 2025-09-05

**Authors:** Suzie Drew, Emily St. John, Michael Bartlett

**Affiliations:** 1 Department of Biology, Portland State University 6685, Portland, Oregon, USA; Montana State University, Bozeman, Montana, USA

**Keywords:** genomes, *Pyrococcus*, archaea, hyperthermophiles

## Abstract

Here, we report the circularized complete genome of *Pyrococcus woesei* DSM 3773^T^ (1,856,505 bp, G + C 40.7%). This genome contains 1,955 protein-coding regions and 46 tRNAs. *P. woesei* is the closest known relative to the archaeal model organism *P. furiosus*.

## ANNOUNCEMENT

*Pyrococcus woesei* was isolated from sulfurous steam vents at a heated marine solfatara off the northern beach of Porto Levante, Vulcano Island, Italy on the Mediterranean Sea (~20 cm sediment depth). Samples were enriched and pure cultures were isolated under anaerobic conditions (1). *P. woesei* is the closest known relative of *P. furiosus*, a model hyperthermophile, but exhibits greater sensitivity to oxidative stress (2). Since their rDNA sequences are identical, *P. woesei* was proposed to be a *P. furiosus* subspecies (3). A subsequent microarray study revealed significant differences between the two genomes, but the full *P. woesei* sequence has not been available for thorough comparative genomics (4).

*P. woesei* DSM 3773^T^ was obtained from DSMZ (Deutsche Sammlung von Mikroorganismen und Zellkulturen GmbH, Braunschweig, Germany). Cells were cultured anaerobically at 85°C for 48 h in sealed tubes containing the rich medium YM5 (seawater medium supplemented with 5 g/L yeast extract and 5 g/L maltose) (5). Genomic DNA was prepared using the Quick-DNA Miniprep Plus Kit (Zymo, Orange, CA).

Short-read library prep was performed using the Illumina Nextera DNA Flex Library Prep Kit. Short-read sequencing was performed with Illumina NovaSeq X Plus, resulting in 929,261 paired-end reads (2 × 151 bp, SeqCenter, Pittsburgh, PA).

Long-read library prep was performed using the Nanopore Rapid Barcoding Kit 96v14 with minimal shearing through tagmentation. Size selection was performed using Beckman Coulter AMPure XP beads. Long-read sequencing was performed with Oxford Nanopore PromethION with flowcell R10.4.1, resulting in 210,415,024 bp of sequence across 99,932 reads (Plasmidsaurus, Monrovia, CA). The N50 was found to be 13,823 using bbmap v39.06 (https://sourceforge.net/projects/bbmap/files/). Base calling was performed through Guppy for PromethION v6.4.8.

Short-read sequence quality was determined using FastQC v0.12.1 (https://www.bioinformatics.babraham.ac.uk/projects/fastqc/). Trimming was performed using FastP v0.23.4 with the parameters --trim_tail1 = 1, --trim_tail2 = 1, --trim_front1 = 20, --trim_front2 = 20, --trim_poly_g --detect_adapter_for_pe, --length_required 35 (6). After trimming, Q20 was 99.02% and Q30 was 95.27%. Long-read sequences were trimmed using Filtlong v0.2.1 (https://github.com/rrwick/Filtlong) using the parameters --min_length 1000, --keep_percent 95.

*De novo* hybrid assembly was performed using long and short reads via Unicycler v0.5.1 with default settings (7). This assembly resulted in a single circularized contig 1,856,505 bp long with a 40.7% G + C content and 122.9× mean short-read coverage, determined using Bowtie2 v2.5.4 (8) and SAMtools v1.21 (9). Circularization overlap was identified in Unicycler v0.5.1 and was not rotated as an origin could not be identified (7). The assembly had an estimated completeness of 99.9% and contamination of 0.36% based on CheckM2 v1.0.2 (10). Proteins and RNAs were predicted using the NCBI Prokaryotic Genome Annotation Pipeline v.6.9 (11), resulting in 1,955 protein coding regions and 46 tRNAs.

One 16S, one 23S, and two 5S rRNA genes were identified. Although the 16S rRNA gene in this assembly showed three single-nucleotide polymorphisms compared to a partial 16S rRNA gene for *P. woesei* (GenBank accession HE654000) (12), it was identical both to the full-length *P. woesei* DSM 3773^T^ 16S rRNA gene (AY519654.1) (3) and to *P. furiosus* DSM 3638^T^ (NR_074375.1) (13).

## Data Availability

This whole genome sequence project has been deposited in DDBJ/ENA/GenBank under the accession no. CP179867. The version described in this paper is the first version, CP179867.1. The BioProject can be found under the accession number PRJNA1216665. Long (SRR33390305) and short read sequences (SRR33390304) are also available in the Sequence Read Archive.
